# State Anxiety Impairs Proactive but Enhances Reactive Control

**DOI:** 10.3389/fpsyg.2018.02570

**Published:** 2018-12-13

**Authors:** Youcai Yang, Tara A. Miskovich, Christine L. Larson

**Affiliations:** ^1^Key Laboratory of Adolescent Health Assessment and Exercise Intervention of Ministry of Education, East China Normal University, Shanghai, China; ^2^Department of Psychology, University of Wisconsin-Milwaukee, Milwaukee, WI, United States; ^3^School of Physical Education and Healthcare, East China Normal University, Shanghai, China

**Keywords:** cognitive control, proactive control, reactive control, AX-continuous performance task, Stroop task, anxiety, working memory

## Abstract

Cognitive control is a construct that prioritizes how we process stimuli and information to flexibly and efficiently adapt to internal goals and external environmental changes. The Dual Mechanism of Control (DMC) theory delineates two distinct cognitive control operations: proactive control and reactive control (Braver, 2012). Anxiety has been posited to differentially affect proactive and reactive control, due to its influence on working memory and attention allocation (Eysenck et al., 2007; Fales et al., 2008). However, no study has yet directly compared the influence of anxiety on proactive and reactive control in the same individuals. In this study, we examined how state anxiety affected proactive control, using the AX-continuous performance task (AX-CPT), and reactive control, using the classic Stroop task. Based on theory and previous investigations, we expected that state anxiety would enhance reactive control but impair proactive control. Consistent with our predictions, we found that state anxiety, induced with a threat of shock manipulation, inhibited proactive control on the AX-CPT test, but increased reactive control in the Stroop task. Anxiety may impair proactive control in contexts requiring goal maintenance by occupying limited working memory capacity, whereas it may enhance reactive control via facilitated attention allocation to threat and engaging the conflict monitoring system to quickly modify behavior.

## Introduction

Cognitive control is defined as the coordination and regulation of thoughts to respond appropriately to salient stimuli in the environment and to maintain focus on goal-directed behavior (Braver, 2012). It includes attention, inhibitory control, working memory, cognitive flexibility, planning, reasoning and problem solving (Chan et al., 2008; Diamond, 2013). Cognitive control is essential for adaptive behavior as it facilitates response to biologically meaningful stimuli, filtering of task-irrelevant information, multitasking and overriding prepotent responses (Miller and Cohen, 2001; Braver, 2012; Enriquez-Geppert et al., 2013). For example, cognitive control can assist you if see a sugar-filled snack you crave but need to minimize your sugar intake for health reasons, or if you are looking for your white car in the parking lot, in which case you need to select your car among all the other white cars while ignoring cars of other colors (Miller and Cohen, 2001). Overall, cognitive control is necessary for us to react to important stimuli quickly (such as avoiding danger) and to override distracting task-irrelevant stimuli to stay on task to achieve internal goals.

The need to balance between focus on goal-directed behavior and responding to important stimuli in the environment requires the ability to flexibly adapt cognitive control to meet current demands (Diamond, 2013). That cognitive control can flexibly shift between goal-directed and stimuli-driven processing suggests that there may be two different cognitive control processes. A recent theory, the dual mechanisms of control (DMC), posits that this balance relies on two different control mechanisms, proactive control and reactive control (Braver, 2012). Proactive control is conceptualized as a goal-driven system, which maintains task-related information in order to bias attention and guide perception and action systems to prepare for the oncoming occurrence of a cognitively demanding event. In contrast, reactive control is defined as stimulus-driven control that is mobilized only as needed. Reactive control has been referred to as a ‘late correction mechanism’ by Braver (2012).

The DMC posits that there is a computational tradeoff between the benefits and costs of proactive and reactive control in order to allow information to be processed efficiently (Braver, 2012). Under proactive control, a goal can be triggered in advance and maintained until the appearance of a salient stimulus, decreasing internal and external interference, flexibly adjusting and facilitating information processing. However, goal maintenance is costly; it consumes resources and occupies capacity-limited working memory stores, which is required for focal attention (Cowan, 2001; McElree, 2001; Oberauer, 2002). In contrast, under reactive control, goal representation is only active after the onset of a stimulus, which is transient and efficient, but the disadvantage is that attention will be easily reallocated whenever there is a triggering event, which can interrupt the execution of a goal.

Anxiety has been shown to impact cognitive control processes, and some theoretical models suggest that anxiety might differentially impact proactive and reactive control (Eysenck et al., 2007; Braver, 2012; Hu et al., 2012). However, little work has examined its specific impact on these two types of cognitive control (Krug and Carter, 2012; Lamm et al., 2013). Anxiety is an aversive emotional and motivational state that occurs during and in anticipation of threatening conditions (Eysenck et al., 2007). State anxiety increases the allocation of attention resources to threat-related stimuli internally and externally, which was initially posited to impair cognitive performance (Sarason, 1988). However, there is also evidence that anxiety does not impair performance (Blankstein et al., 1990, 1989). Eysenck et al. (2007)’s Attentional Control Theory (ACT) attempted to reconcile this. They proposed that anxiety affects processing efficiency, resulting in the need for compensatory processes to spare performance (Eysenck et al., 2007). Anxiety is thought to impair processing efficiency by restricting the capacity of working memory; and indeed, high anxiety subjects have been found to have less capacity than those low on anxiety (Darke, 1988; Stout and Rokke, 2010; Moran, 2016). The goal maintenance necessary for proactive control depends on working memory and goal-directed attentional control (Duncan et al., 1996; Kane and Engle, 2002; Braver, 2012); thus proactive control is posited to be impaired by anxiety (Moser et al., 2013). In addition, anxiety is associated with decreased attentional control (Coombes et al., 2009), and impairment of inhibition (Eysenck et al., 2007; White et al., 2011). This, in turn, would require individuals to rely more on reactive control through stimulus-driven attention (Eysenck et al., 2007). Consistent with this framework, in a neuroimaging study of working memory, Fales et al. (2008) found that a negative mood induction led to a shift from sustained to transient activation in working memory regions. Since sustained activity subserves proactive control and transient activity reactive control (Braver, 2012), these findings suggest that anxiety is associated with reduced proactive and enhanced reactive control.

Even though some initial evidence suggests anxiety differentially affects proactive and reactive control, more investigation is needed. The differential effect of state anxiety on proactive versus reactive control has not yet been directly compared in the same individuals. The aim of this study was to examine how proactive and reactive control are affected under state anxiety. To test this we administered tasks that have been used extensively to assess proactive control, the AX Continuous Performance Task (AX-CPT) (Braver et al., 2001, 2005; Locke and Braver, 2008; Paxton et al., 2008), and reactive control, the Stroop task (Botvinick et al., 2001; Stout and Rokke, 2010; Gonthier et al., 2016; Kalanthroff et al., 2018), both under threat of shock and safety.

During the AX-CPT, participants respond to a probe based on the identity of a preceding cue, with a brief delay separating cue and probe. Cue and probe stimuli are sequentially presented letters. Participants make a target response when the see the target probe, which is the letter “X,” but only when it follows the cue letter “A” (AX target trial). Non-target responses are required for any other sequence of paired letters, including AY trials (A followed by any letter except X), BX trials (any letter but A precedes X), and BY trials (any non-A cue followed by any non-X probe). Target trials (AX trials) are presented with high frequency compared to non-target trials. Thus, during these non-target trials, participants must inhibit the prepotent response to the probe “X” (Paxton et al., 2008). Use of proactive control is evident when participants maintain the cue information during the delay to inform response to the probe. Thus, proactive control serves to enhance performance on BX trials, as participants maintaining the B cue are prepared not to respond to the X as a target. In contrast, proactive control results in worse performance on AY trials, as participants prepare to respond (incorrectly) to an anticipated X target (Braver et al., 2007; Braver, 2012; Gonthier et al., 2016). Moreover, manipulations that enhance proactive control enhance BX and impair AY performance (Gonthier et al., 2016). Together these data indicate that BX and AY performance are established assays of proactive control.

To measure reactive control we used the Stroop task (Stroop, 1935). The classic Stroop tasks instructs participants to name the color of ink or font that color words are presented in. When a person is instructed to name the colors of the ink or font that the word “GREEN” is presented in (e.g., green or red ink), much more time is required when the color of the ink is incongruent with the meaning of the word (e.g., “green” presented in red ink), compared to when the color of ink matches the printed word. We altered the Stroop task to increase the percentage of congruent (70%) versus incongruent trials (30%). Increasing the number of congruent trials boosts the tendency toward making the prepotent word-reading response, thus relaxing proactive control and increasing the reliance on reactive control in the incongruent trials (Stout and Rokke, 2010).

We hypothesized that when under threat of unpredictable shock, participants would demonstrate impaired proactive control, as indexed by poorer performance on BX trials and improved performance on AY trials in the AX-CPT, and enhanced reliance on reactive control, operationalized as enhanced performance on incongruent trials in the Stroop task.

## Materials and Methods

### Participants

This study was approved by University of Wisconsin-Milwaukee IRB. Seventy-three participants aged 18–35 were recruited from the University of Wisconsin-Milwaukee. All participants were granted 2 h of course extra credit and one $10 gift card. All participants had normal color vision. The sequences of the AX-CPT and Stroop tasks were counterbalanced across participants. Ten participants were excluded because of technical problems with shock delivery. Two participants were excluded because less than 50% of trials in the Stroop or AX-CPT task were answered correctly. One participant had a greater than a 50% error rate in the AX-CPT and another in the Stroop task. They were dropped from both tasks so the samples were the same across task. The final sample consisted of 61 participants (52 F, 9 M; Mean age = 21.4 (4.1); 42 Caucasian (68.85%).

### Threat of Shock Manipulation

Before the Stroop and AX-CPT tasks, participants underwent a shock workup procedure to establish a level of shock that was ‘painful but can tolerable’ and to be used throughout the experiment. The workup and the task shocks were both delivered to the same ankle. Shocks were delivered using Psychlab’s SHK1 Pain Stimulation Shocker (Contact Precision Instruments, Cambridge, MA, United States). The electrical shock was a constant current at the individually determined level delivered via an electrode placed on the outside of the participant’s right or left ankle for 500 ms. Stimulation was delivered via two sensors placed approximately 2 in. above the right or left ankle (using double-sided tape and conductive gel). For the shock workup participants were told that they would receive a mild electric shock and would be asked to rate it from 1 to 10, 1 being “I didn’t feel anything,” and 10 being “painful, but tolerable.” The experimenter increased the shock level gradually until the participant rated the shock a 10. The goal was to determine a level that the participant subjectively rated as a 10: painful, but tolerable. Once that shock level was established, shock was set at that level for the duration of task; the participant could increase or decrease the level at any point in the study if they became too uncomfortable or habituated to the shock. Two participants increased their shock level during a break because they habituated to the shock. Their data were included in analyses. All other participants maintained their initial shock level.

### Stroop Task Design and Procedures

**FIGURE 1:**
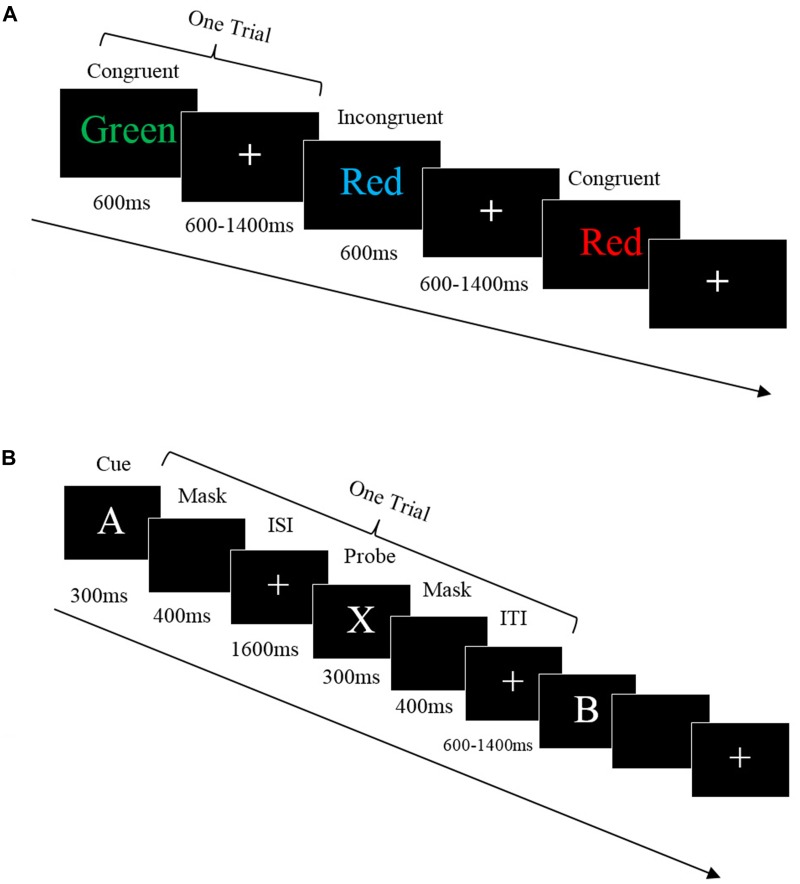
**(A)** Stroop task. Each trial started with a color word shown on the screen for 600 ms, followed by a white fixation cross shown on the screen varying from 600 to 1,400 ms. The participants were asked to respond to the color of the words but not the meaning by pressing the same color button on the keyboard. There were two word conditions: Congruent and Incongruent. In congruent condition, the word reading and color naming were the same whereas the incongruent are not. **(B)** AX-CPT task. Each trial started when a white cue appeared on screen for 300 ms then masked for 400 ms. A fixation appeared on the screen for 1,600 ms, then the target was presented for 300 ms, then masked for 400 ms. The ITI varied from 600 to 1,400 ms, then the next trials started. Participants had 2,100 ms to respond.

The Stroop task was modified from the classic color-word Stroop task (Stroop, 1935). Each trial included a color word shown on the screen for 600 ms, followed by a white fixation cross varying from 600 to 1,400 ms. Participants were asked to respond to the color of the text the word was displayed in, but not the meaning of the word, by pressing the same color button on the keyboard as accurately and quickly as possible. There were two word conditions: Congruent and Incongruent. In the congruent condition, the words ‘GREEN,’ ‘RED,’ and ‘BLUE’ were presented in their own color to maintain congruence of word reading and color naming. In the Incongruent condition, the words ‘GREEN,’ ‘RED,’ and ‘BLUE’ were presented in different colors from their meaning to cause interference. For example, when the word ‘GREEN’ was shown on the screen in the color red the participant should press the red button on the keyboard (see Figure 1A).

There were two state anxiety conditions: safe and threat of shock. For the safe condition, there was a 30 pixel wide blue border around the edge of the screen and participants were explicitly told that they would not receive any shocks. For the shock conditions, the 30 pixel wide border was red and participants were explicitly told that they might receive shock(s) on their ankle at any time.

The Stroop task consisted of six blocks, with three safe and three shock blocks (a total of 150 trials in each condition, shock and safe), in alternating order. The condition of the first block, safe or threat, was randomly determined. In each of six blocks, there were 35 congruent trials (70% of trials) and 15 incongruent trials (30%), with the trial order randomly assigned. During the shock block, participants might receive one, two or three electrical shock(s). After each block, participants rated their current anxiety level by pressing a button between 1 (low anxiety) and 7 (high anxiety). The within-block timing of shock administration was randomized.

### AX-CPT Task Design and Procedures

The AX-CPT task consisted of continuous trials with a single letter presented on the computer, with each letter requiring a button press response from the participant. In each trial, a letter (cue) was displayed and followed by its paired letter (probe), which together comprised a Cue-Probe sequence. There were four Cue-Probe sequence trial types: AX, AY, BX, and BY. The ‘A’ represented the target cue while ‘B’ represented the non-target cue, ‘X’ represented the target probe while the ‘Y’ represented the non-target probe. During the AX target trials, only the letters A and X were presented. However, in addition to A, B, X, and Y the non-target trials (AY, BX, and BY) also included the letters E, F, G, J, M, P, Q, R, S, U, and V. Each letter in the sequence was used only as a cue or as a probe. The probe letter never served as the cue for the next trial. The participants were instructed to respond to each letter (cue and probe) by pressing button ‘1’ (Yes, the target sequence completed) or ‘2’ (No, the target sequence did not complete). That is, participants only pressed ‘1’ when letter X (probe) followed the letter A (cue), which completed a target cue-probe sequence. Other than this, participants were instructed to press ‘2’ to any cues and probe (e.g., B-X, A-G, M-Q). Each trial started when a white cue appeared on the screen for 300 ms followed by a blank screen for 400 ms (see Figure 1B). After a fixation appeared on the screen for 1,600 ms, the target appeared on the screen for 300 ms then was masked for 400 ms. The ITI varied from 600 to 1,400 ms. Participants had 2,100 ms to make a response.

To create a tendency to rely on proactive control, we attempted to instill a prepotent response to respond to the X (with a ‘1’ button press) by presenting the AX target trial type more frequently (70% of trials) than the non-target trial types: 10% each for AY, BX, and BY.

Trials were presented under both threat of shock and safe conditions. The shock procedure was the same as the Stroop task. The safe block had a 30 pixel wide blue border around the edge of the screen, whereas a 30 pixel wide red border signaled that the participant may receive a shock at any time. Participants were also explicitly told whether they would potentially receive any shocks or not before each block.

The AX-CPT task consisted of 10 blocks, with five safe and five shock blocks, in alternating order. The condition of the first block, safe or threat, was randomly determined. In each block there were 40 trials, including 28 AX, 4 AY, 4 BX, and 4 BY trials. All trial types were presented in a random order. During the five shock blocks participants received between 0 and 3 shocks (one block each of 0, 1, and 3 shocks, 2 blocks with 2 shocks). The order of these shock blocks was randomly assigned among shock block positions. The within-block timing of shock administration was randomized.

Before the experimental trials, participants conducted a practice block. After each block, subjects were asked to rate their anxiety on a 7-point scale (1 = low, 7 = high).

### Data Analysis

#### Stroop

2.56% trials were excluded from analysis due to lack of response, 0.18% trials were excluded because the RT was less than 200 ms, 1.91% trials were excluded because the shock occurred during this trial, and 0.76% trials were excluded because of RT longer than 3 standard deviations from the mean for each participant. In total, 5.41% of trials were dropped.

All accuracy and RT data were examined using a 2 (Condition: Safe vs. Threat) × 2 (Trial Type: Congruent vs. Incongruent) repeated measures ANOVA. A series of paired *t*-tests were used to decompose significant interactions.

#### AX-CPT

The dependent variables were accuracy and reaction time for responses to the probe letters. Only trials for which participants responded correctly to the cue were analyzed. 2% of trials were excluded from analysis because the shock occurred, 4.28% trials were excluded because of incorrect or no response to the cue. The Shapiro–Wilk test of normality revealed that the RT data for the AX (*p* = 0.005), BX (*p* < 0.001) and BY (*p* < 0.001) trial types in the safe condition were not normally distributed, and that the RTs for the AX (*p* = 0.057), BX (*p* < 0.001) and BY (*p* < 0.001) trial type in threat condition were also not normally distributed. Therefore, as has been done in other AX-CPT studies (Barch et al., 2004; Lopez-Garcia et al., 2016), the median of the RT of each participant for each condition and trial type was used for the RT analysis to reduce the influence of outlier responses. AX-CPT accuracy and RT were examined using a 2 (Condition: Safe vs. Threat) × 4 (Trial Type: AX, AY, BX, and BY) repeated measures analysis of variance (ANOVA). Bonferroni-corrected *post hoc* comparisons were used to follow-up on significant interactions or effects of Trial Type.

In the repeated measure ANOVAs, if the Mauchly’s test of sphericity assumption was violated, the Greenhouse–Geisser epsilon was used to correct the degrees of freedom.

## Results

### Anxiety Ratings

**FIGURE 2:**
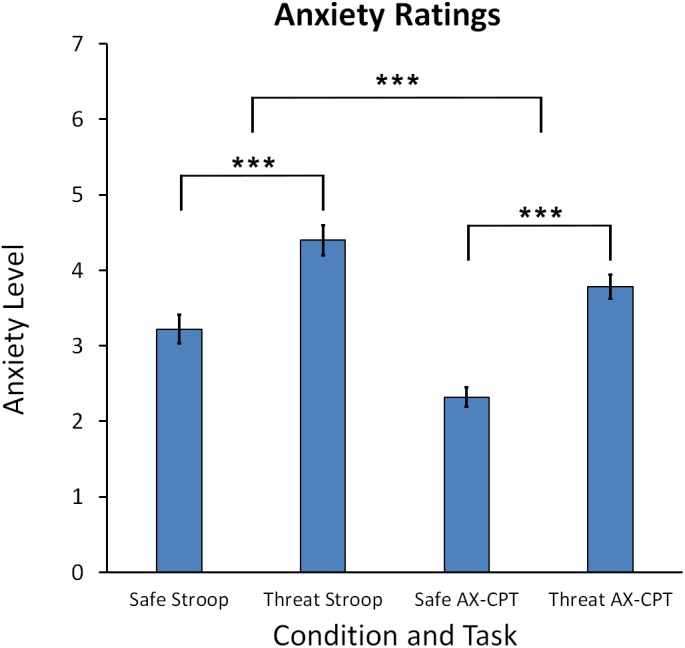
Mean anxiety ratings for the Stroop task and AX-CPT task. Error bars represent the standard error of the mean. Asterisks represents a significant difference. ^∗∗∗^*p* < 0.001.

Anxiety ratings taken at the end of each block indicated that participants felt more anxious during the threat of shock compared to safe blocks for both tasks. A Task (Stroop, AX-CPT) × Condition (Threat, Safe) repeated measures ANOVA indicated a main effect for Condition, *F*(1,60) = 88.79, *p* < 0.001, reflecting higher self-reported anxiety for threat compared to safe blocks. Cohen’s *d* for the comparison of threat vs. safe was *d* = 0.399 for the Stroop task and *d* = 0.642 for the AX-CPT (see Figure 2). In addition, there was also a significant main effect for Task, *F*(1,60) = 30.32, *p* < 0.001, and a Task × Condition interaction, *F*(1,60) = 4.387, *p* = 0.04, which showed that anxiety ratings were higher for the Stroop than the AX-CPT task, *p* < 0.001, $\eta_{p}^{2}$ = 0.336, and that the increase in anxiety for threat vs. safe was greater for the AX-CPT than the Stroop, *t*(60) = 2.10, *p* = 0.04.

### Stroop

#### Accuracy

**FIGURE 3:**
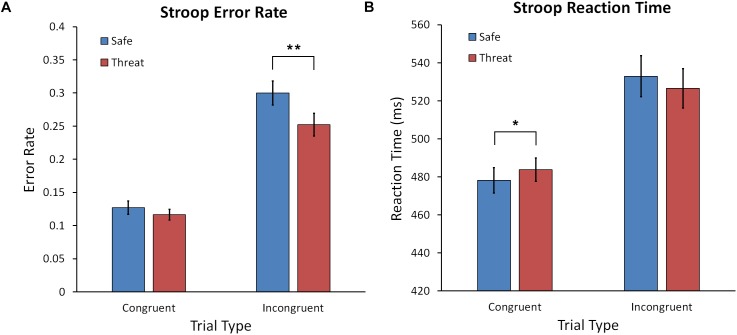
**(A)** Mean error rate for the Stroop task for the safe and threat of shock conditions for congruent and incongruent trials. **(B)** Mean reaction time for the Stroop task for safe and threat of shock conditions across trial types. Error bars represent the standard error of the mean. Asterisks represents a significant difference. ^∗^*p* < 0.05; ^∗∗^*p* < 0.01.

A Condition (Safe, Threat) × Trial Type (Congruent, Incongruent) repeated measures ANOVA yielded a significant interaction, *F*(1,60) = 4.246, *p* = 0.044, $\eta_{p}^{2}$ = 0.007, main effect of Condition, *F*(1,60) = 9.404, *p* = 0.003, $\eta_{p}^{2}$ = 0.135, and Trial Type, *F*(1,60) = 87.436, *p* < 0.001, $\eta_{p}^{2}$ = 0.593 (see Figure 3A). Performance for the incongruent trial type was poorer than congruent for both safe and threat conditions. However, as reflected by the interaction, threat affected performance differently for congruent and incongruent trials. For incongruent trials, participants made fewer errors under threat of shock than during safety, *t*(60) = 3.002, *p* = 0.004, Cohen’s *d* = 0.388. However, error rates did not differ between threat and safe for congruent trials, *t*(60) = 0.980, *p* = 0.331, Cohen’s *d* = 0.127. This suggests that anxiety facilitated performance on the incongruent trials, in which reactive control is required to prevent engaging in the dominant word reading response.

#### Reaction Time

The identical Condition × Trial Type ANOVA was conducted with RT as the dependent variable. This ANOVA yielded a significant interaction, *F*(1,60) = 6.362, *p* = 0.014, $\eta_{p}^{2}$ = 0.096, and main effect of Trial Type, *F*(1,60) = 69.855, *p* < 0.001, $\eta_{p}^{2}$ = 0.538 (see Figure 3B). As expected RTs were faster for the easier congruent trials compared to incongruent trials. Following up on the significant interaction revealed that RTs were slower for congruent trials during shock compared to safe conditions, *t*(60) = 2.064, *p* = 0.043, Cohen’s *d* = 0.267.

### AX-CPT

#### Accuracy

**FIGURE 4:**
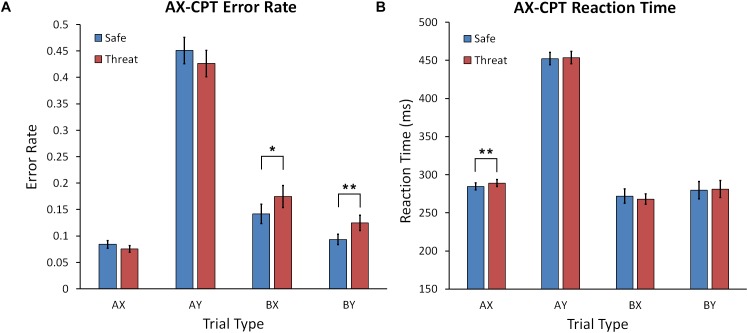
**(A)** Mean error rate for the AX-CPT task for safe and shock conditions across trial types. **(B)** Mean reaction time for the AX-CPT task for safe and shock conditions for all trial types. Error bars represent the standard error of the mean. Asterisks represents a significant difference. ^∗^*p* < 0.05; ^∗∗^*p* < 0.01.

A Condition (Safe, Threat) × Trial Type (AX, AY, BX, and BY) repeated measures ANOVA yielded a significant interaction, *F*(2.469,148.134) = 4.675, *p* = 0.004, $\eta_{p}^{2}$ = 0.072, and main effect of Trial Type, *F*(1.803,108.168) = 127.966, *p* < 0.001, $\eta_{p}^{2}$ = 0.681, but no main effect of Condition (see Figure 4A). *Post hoc* comparisons across threat and safe conditions (Bonferroni corrected) showed that the error rate for the AX trial type was significantly lower than for AY (*p* < 0.001), and BX (*p* < 0.001), but not BY (*p* = 0.088). Participants also made more errors during AY than BX (*p* < 0.001) and BY (*p* < 0.001) trials. The error rate for BX was also higher than BY (*p* < 0.001). Following up on the significant interaction, we found that the error rate was higher in the threat compared to safe condition for the BX, *t*(60) = 2.109, *p* = 0.039, Cohen’s *d* = 0.272, and BY, *t*(60) = 2.690, *p* = 0.009, Cohen’s *d* = 0.347, trial types. There was a trend for fewer errors under threat of shock in the AX condition, *t*(60) = 1.906, *p* = 0.061, Cohen’s *d* = 0.246. No significant error rate difference was found between threat and safe in the AY condition, *p* = 0.189.

#### Reaction Time

A Condition (Safe, Threat) × Trial Type (AX, AY, BX, and BY) ANOVA was calculated with RT as the dependent variable. There was a significant main effect of Trial Type, *F*(2.085,123.007) = 499.809, *p* < 0.001, $\eta_{p}^{2}$ = 0.894, but no main effect for Condition, *F*(1,59) = 0.530, *p* = 0.470, $\eta_{p}^{2}$ = 0.009, or Condition × Trial Type interaction, *F*(2.085,123.007) = 499.809, *p* = 0.933, $\eta_{p}^{2}$ = 0.002 (see Figure 4B). *Post hoc* comparisons across threat and safe conditions (Bonferroni corrected) showed that RT for the AX trial type was faster than AY (*p* < 0.001), slower than BX (*p* = 0.004), and did not differ from BY (*p* = 0.090). RT for AY was also slower than BX (*p* < 0.001) and BY (*p* < 0.001). RT for BX was not significantly faster than BY (*p* = 0.602). Despite the lack of interaction, we did conduct *post hoc* comparisons to test our *a priori* hypotheses. There was no significant difference between threat and safe during the AY, BX, or BY conditions, *F*(1,59) = 0.530, *p* = 0.470, $\eta_{p}^{2}$ = 0.002. We did find that RT was significantly slower during threat than safe for AX trials, *t*(60) = 3.336, *p* = 0.001, Cohen’s *d* = 0.431.

### Correlations Between Tasks

In order to explore whether proactive and reactive control under threat (vs. safety) were related, we correlated RT and error rate for threat minus safety across the two tasks. We correlated all conditions but our primary focus was on correlations between the primary indices of reactive control (Stroop incongruent trials) and proactive control (AX-CPT BY and AY trials). Raw RT was highly correlated across all conditions, reflecting strong global individual differences in RT. Therefore, prior to calculating the RT correlations we calculated modified z-scores (which use the median rather than the mean) in order to allow for meaningful inter-individual correlations. Holm-Bonferroni correction was applied. No cross-task correlations survived correction for either error rate or RT. A full presentation of these threat minus safe correlations (both across and within task) can be found in Supplementary Tables 1, 2.

In addition to the threat minus safe cross task correlations, for completeness we also correlated all conditions across both tasks for both error rate and RT (using the modified *z*-scores). For RT there were no significant correlations surviving Holm-Bonferroni correction (and only one significant correlation prior to correction) across all threat and safe conditions between the Stroop and AX-CPT trial types. For error rate, there were no correlations involving AX-CPT BX trials or Stroop Incongruent trials. We did find that more errors on AY trials was correlated with making more errors on both Stroop incongruent and congruent trials for both threat and safe conditions (*p*s for all eight correlations <0.01, with the exception of Threat AY and Threat Incongruent for which *p* = 0.12). This likely reflects that the overall demands of AY trials (inhibiting the prepotent response to the A cue) are most similar to the demands required by the Stroop. Correlations between all conditions across and with task are presented in Supplementary Tables 3–8. Overall, we did not find evidence of relations between proactive and reactive control, regardless of the presence of threat.

## Discussion

We sought to compare how state anxiety differentially impacts two distinct forms of cognitive control. Using well-established assays of proactive and reactive control, we found support for the hypothesis that state anxiety impairs proactive control but enhances reactive control. Reactive control was assessed using a Stroop task modified to increase reliance on reactive control during incongruent trials. As predicted, we found that threat of shock led to better performance on these reactive control-reliant incongruent trials. Proactive control was measured using performance on BX and AY trials of the AX-CPT. As expected, threat of shock impaired performance on BX trials in the AX-CPT, a condition for which optimal performance depends on maintenance of cue information to inhibit a false alarm to the X. The introduction of state anxiety appeared to dampen this proactive control mechanism, resulting in more false alarms. We also predicted that state anxiety would improve performance on AY trials, in which proactive control can actually harm performance by enhancing the prepotent tendency to respond to any letter following an A as a target. While the means were numerically consistent with improved AY performance under anxiety, this difference was not significant. Overall, the findings from these two tasks indicate that anxiety enhances reactive and impairs proactive control, an effect which has not previously been demonstrated by directly comparing the influence of state anxiety on these two types of control in the same individuals.

As noted, the Stroop task served as our index of reactive control. The Stroop task does require proactive control, in that contextual information or trial-by-trial maintenance is required to make a response. However, modifying the Stroop task to increase the percentage of congruent trials, as we did here, serves to relax proactive control and cause greater reliance on reactive control (Botvinick et al., 2001; Stout and Rokke, 2010; Gonthier et al., 2016; Kalanthroff et al., 2018). Thus, in order to respond correctly on the rare incongruent trials individuals had to engage reactive control to avoid word reading, resulting in an incorrect response (Botvinick et al., 2001). As we hypothesized, state anxiety facilitated reactive control; participants made fewer errors on incongruent trials under threat of shock compared to safe conditions. This result is consistent with a similar Stroop finding in which threat of shock slowed responding during neutral Stroop trials but facilitated responding on incongruent trials (Hu et al., 2012). Jointly, the increased proportion of congruent trials and introduction of anxiety likely both served to dampen proactive control, resulting in relatively greater reliance on reactive control. Indeed, earlier studies using equal numbers of congruent and incongruent trials found that individuals made more errors during incongruent trials under threat of shock or other stress (Hochman, 1967; Pallak et al., 1975) (but see Hu et al., 2012). This suggests that anxiety specifically facilitates reactive control when proactive control has been relaxed.

The facilitation of reactive control under threat could be interpreted as consistent with the attention narrowing hypothesis, which posits that anxiety enhances attention on salient stimuli (Callaway and Dembo, 1958; Callaway, 1959; Easterbrook, 1959; Eysenck and Calvo, 1992; Chajut and Algom, 2003; Eysenck et al., 2007). Relatedly, threat may facilitate reactive control by enhancing the activity of anterior cingulate cortex (ACC)-driven conflict monitoring system (Egner and Hirsch, 2005; Kerns et al., 2005). The ACC is a critical node in the conflict monitoring system, which is responsible for overriding prepotent responses, (Botvinick et al., 2004), as is necessary in the rare incongruent trials in our study (Carter et al., 2000). fMRI and EEG studies have shown that during high conflict correct responses, the ACC subserves adaptive conflict monitoring, including error detection and behavioral correction, and it is the only area that shows greater activation when behavior is subsequently adjusted after conflict is detected (Carter et al., 1998; Garavan et al., 2002). High anxious individuals have previously been shown to exhibit stronger EEG signatures of conflict monitoring (Schmid et al., 2015). In an EEG study using the numeric Stroop test, those high on math anxiety did not initially show conflict adaptation, but over time were able to adapt to conflict by boosting ACC engagement (Suárez-Pellicioni et al., 2014). This is consistent with the idea that anxious individuals exert control in a reactive way. Another ACC-dependent event-related potential involved in conflict monitoring, the error-related negativity (ERN) (Falkenstein et al., 1991; Gehring et al., 2018), has been shown to be amplified in response to punishment (Riesel et al., 2012) and among anxious individuals (Hajcak et al., 2004; Moser et al., 2011; Weinberg et al., 2012; Zambrano-Vazquez and Allen, 2014). Together, these findings indicate that anxiety, both state and trait, is associated with heightened engagement of the ACC conflict monitoring system, likely to facilitate adaptive reactive control in the face of limited proactive control resources.

We did not find any effect of state anxiety on reaction times for incongruent trials. If state anxiety enhances reactive control, then it might be expected that RT for the incongruent trial type may be slowed, allowing for time to engage reactive control in the face of conflict (Kalanthroff et al., 2018). However, our findings suggest that state anxiety facilitated accurate performance and that this did not come at the expense of a longer response time.

For proactive control, overall the pattern of findings across the four trial types in the AX-CPT task was largely consistent with previous work (Cohen et al., 1999; Barch et al., 2001; Lopez-Garcia et al., 2016), with subjects performing best on AX and worse on BX and AY trials. We focused on the impact of state anxiety on BX and AY trials (Gonthier et al., 2016). The attention and inhibitory functions in the AX-CPT test are subserved by an internal representation of context information, and rely on working memory regions of the dorsolateral prefrontal cortex (dlPFC) (Braver, 2012). Anxiety is thought to impair processing efficiency required for such inhibitory tasks by restricting the capacity of working memory (Darke, 1988) and increasing the allocation of these resources to threat-related stimuli internally and externally (Sarason, 1988; Amir et al., 1998; Bar-Haim et al., 2007). Braver (2012) has posited that sustained dlPFC activity, as is evident in working memory (Braver and Cohen, 1999; Fales et al., 2008), subserves proactive control. Consistent with the hypothesis that anxiety impairs proactive control, anxious individuals show reduced sustained activity of the dlPFC during a working memory task (Fales et al., 2008). This decreased maintenance of dlPFC activity would lead to deficits in maintaining contextual information needed to maintain focus on task-relevant responses in the face of salient distracting information.

Following this logic, we expected performance on BX trials to be impaired under anxiety, as they require more working memory maintenance to prevent a false alarm response to the “X.” Indeed, we found the error rate for BX was higher in the threat than safe condition. This finding suggests that anxiety impaired the override of the prepotent response to the probe X, which requires maintenance of the contextual information provided by the B cue during the delay. Based on previous work, it is likely that state anxiety occupied limited working memory resources, thus impairing maintenance of this contextual information, adversely impacting proactive control. Threat of shock has previously been shown to impair working memory performance (Shackman et al., 2006; Vytal et al., 2012), which has been suggested to be due to competition of sensory perceptual and cognitive resources (Robinson et al., 2013). More specifically, impairments may be explained by the occupation of limited working memory capacity by anxious cognitions, resulting in worse performance on the working memory task (Eysenck and Calvo, 1992; Vytal et al., 2012, 2013). This is consistent with work showing that low anxious, but not high anxious participants, are able to rely on dlPFC-dependent proactive control (whereas anxious individuals are more dependent on reactive control) (Schmid et al., 2015). Our finding that BX performance was impaired under threat of shock is consistent with these previous studies highlighting the adverse impact of anxiety on proactive control and working memory processes needed for effective proactive control.

We had also predicted that threat of shock would improve performance on AY trials, as reliance on proactive control, or maintaining the ‘A’ cue increases the likelihood of expecting a subsequent ‘X’ and thus making an error. However, we did not find a difference in performance on AY trials for threat compared to safe conditions. It is possible that this lack of finding is due to the high error rate in general for the AY trials, which may have prevented detection of differences between conditions. In addition to threat’s impact on BX trials we also somewhat unexpectedly found that threat similarly impacted BY performance, such that more errors were made in threat compared to safe condition. It is not clear why threat impacted BY performance as BY is not thought to tap proactive control.

While not related to our core question, we did find that threat of shock slowed RT during both congruent trials on the Stroop and AX trials. In both cases these trials were presented with high frequency (70%) establishing a more automatic prepotent response. Across tasks we find that state anxiety compromised speed in performing these simplest task conditions. This suggests that state anxiety may slow response speed in these low control conditions by relocating attention to potential threat. This is consistent with visual search data showing that reaction time is slowed when searching a display of all threat stimuli compared to all non-threat stimuli (Larson et al., 2007).

Overall our findings are consistent with the ACT (Eysenck et al., 2007) which posits that anxiety impairs efficient functioning of the goal directed attentional system and enhances processing by the stimulus-driven attentional system. Thus, attentional control is decreased, but attention to threat-related stimuli is enhanced. ACT suggests the anxiety occupies the limited working memory capacity with threat-related information, both task-relevant and irrelevant. This leads to low central executive performance, especially inhibition, but high performance on conflict monitoring. In other words, anxiety may utilize more working memory resources on reallocation of attention to task-unrelated stimuli, which serves to enhance reactive control but impair proactive control, as has been observed in studies such as those cited above (Eysenck et al., 2007; Fales et al., 2008; Hu et al., 2012). This is also consistent with the DMC theory that proactive and reactive control shift according to task demands of environment, ideally to adaptively engage in goal directed behavior. However, when the environment enhances state anxiety, under high working memory load proactive control is impaired and individuals may rely more on reactive control, which may lead to poorer performance on tasks requiring goal-maintenance. According to the Attention Control Theory this same anxious state enhances stimulus-driven attention, which facilitates DMC reactive control, and allows for quick modifications of behavior such as that seen on the incongruent Stroop trials in our study.

## Conclusion

We found that state anxiety differentially impacted proactive and reactive cognitive control. State anxiety enhanced performance in a Stroop task designed to make individuals rely on reactive control, potentially by facilitating the conflict monitoring system, enabling modification of behaviors according to environmental changes. Enhanced reactive control under threat may have adaptive functions in altering ongoing behavior to respond appropriately to potential threats. In contrast, state anxiety impaired performance in situations requiring proactive control. Anxious cognitions may compete with goal maintenance demands for limited working memory capacity, which adversely impacts performance on tasks relying on proactive control. The processing of task irrelevant information, particularly potential threat, may be adaptive if threat is real and imminent, but in other cases interferes with execution of ongoing task goals, and impairs performance. The interesting additional finding of state anxiety slowing of responses in simple task conditions also supports the idea that potential threat occupies limited resources and impacts task performance. In sum, state anxiety differentially impacts reactive and proactive control, in ways that reflect adaptive responding to potential threats in the environment, but that may also compromise performance on more complex tasks that require proactive control for optimal performance.

## Ethics Statement

This study was carried out in accordance with the recommendations of the University of Wisconsin-Milwaukee Institutional Review Board with written informed consent from all subjects. All subjects gave written informed consent in accordance with the Declaration of Helsinki. The protocol was approved by the University of Wisconsin-Milwaukee Institutional Review Board.

## Author Contributions

YY contributed to the study design, data collection, analyses, and manuscript drafting. TM contributed to the task design and manuscript review. CL contributed to the task design, manuscript development and review, and supervised the conduct of the project.

## Conflict of Interest Statement

The authors declare that the research was conducted in the absence of any commercial or financial relationships that could be construed as a potential conflict of interest.
