# Supplementary material for: State Anxiety Impairs Proactive but Enhances Reactive Control
Source: Front Psychol. 2018 Dec 13;9:2570. doi: 10.3389/fpsyg.2018.02570 (PMC6300490; doi:10.3389/fpsyg.2018.02570)
Supplement: Supplementary file 1 [file Data_Sheet_1.PDF]

Supplementary Materials

In order to explore whether proactive and reactive control under threat (vs. safety) were related we correlated RT and error rate for threat minus safety across the two tasks. We correlated all conditions but our primary focus was on correlations between the primary indices of reactive control (Stroop incongruent) and proactive control (AX-CPT BY and AY) trials. Raw RT was highly correlated across all conditions, reflecting strong global individual differences in RT. Therefore, prior to calculating the RT correlations we calculated modified z-scores (which use the median rather than the mean) in order to allow for meaningful inter-individual correlations. Holm-Bonferroni correction was applied. No cross-task correlations survived correction for either error rate or RT. A full presentation of these threat minus safe correlations (both across and within task) can be found in below in Tables 1 and 2.

In addition to the threat minus safe cross task correlations for completeness we also correlated all conditions across both tasks for both error rate and RT (using the modified z-scores). For RT there were no significant correlations surviving Holm-Bonferroni correction (and only one significant correlation prior to correction) across all threat and safe conditions between the Stroop and AX-CPT trial types. For error rate, there were no correlations involving AX-CPT BX trials or Stroop Incongruent trials. More errors on AY trials was correlated with making more errors on both Stroop incongruent and congruent trials for both threat and safe conditions (*p*s for all eight correlations < .01, with the exception of Threat AY and Threat Incongruent for which p = .12). This likely reflects that the overall demands of AY trials (inhibiting the prepotent response to the A cue) are most similar to the demands required by the Stroop. Correlations between all conditions across tasks for RT and error rate are presented below in Tables 3 and 4. Tables 5 and 6 present RT correlations within tasks (Table 5 for AX-CPT and Table 6 for Stroop). Finally, Tables 7 and 8 present within task error rate correlations for the AX-CPT and Stroop, respectively. Overall, we did not find evidence of relations between proactive and reactive control, regardless of the presence of threat.

Table 1. RT correlations across tasks and conditions, Threat minus Safe difference scores.

		**AX-CPT**			
		**AX**	**AY**	**BX**	**BY**
**Stroop**	**Congruent**	.05	.03	.06	.27*
	**Incongruent**	.05	-.03	.08	.23

**p* < .05, ***p* < .01, ****p* < .001

Table 2. Error rate correlations across tasks and conditions, Threat minus Safe difference scores.

		**AX-CPT**			
		**AX**	**AY**	**BX**	**BY**
**Stroop**	**Congruent**	-.05	-.05	.25	.02
	**Incongruent**	.18	.01	-.16	-.05

**p* < .05, ***p* < .01, ****p* < .001

Table 3. RT correlations across tasks and conditions.

			AX-CPT							
			Safe				Threat			
			AX	AY	BX	BY	AX	AY	BX	BY
Stroop	Safe	Congruent	.20	.02	-.01	.03	-.09	-.04	-.16	-.05
		Incongruent	-.05	.06	.06	.22	-.14	-.01	.04	.08
	Threat	Congruent	.20	.03	-.10	-.30*	.12	.09	-.14	-.14
		Incongruent	.04	-.06	-.09	-.24	.14	.03	-.07	-.11

*p < .05, **p < .01, ***p < .001

Table 4. Error rate correlations across tasks and conditions.

			AX-CPT							
			Safe				Threat			
			AX	AY	BX	BY	AX	AY	BX	BY
Stroop	Safe	Congruent	.22	.47**	-.07	.09	.25*	.38**	.04	.23
		Incongruent	.10	.35**	.12	.02	.03	.33*	-.01	.07
	Threat	Congruent	.23	.51**	.01	.20	.23	.57**	.11	.37**
		Incongruent	.06	.31*	.15	.02	.07	.20	.02	.05

*p < .05, **p < .01, ***p < .001

Table 5. RT correlations across conditions in the AX-CPT.

		Safe				Threat			
		AX	AY	BX	BY	AX	AY	BX	BY
Safe	AX								
	AY	-.07							
	BX	-.20	.07						
	BY	-.28*	-.03	.77**					
Threat	AX	.37**	-.22	-.45**	-.50**				
	AY	.02	.47**	-.03	-.01	-.02			
	BX	-.31*	-.07	.60**	.55**	-.28*	-.22		
	BY	-.27*	-.07	.71**	.80**	-.43**	.00	.57**	

*p < .05, **p < .01, ***p < .001

Table 6. RT correlations across conditions in the Stroop.

		Safe		Threat	
		Congruent	Incongruent	Congruent	Incongruent
Safe	Congruent				
	Incongruent	.68**			
Threat	Congruent	-.28*	-.76**		
	Incongruent	-.70**	-.99**	.75**	

*p < .05, **p < .01, ***p < .001

Table 7. Error rate correlations across conditions in the AX-CPT.

		Safe				Threat			
		**AX**	**AY**	**BX**	**BY**	**AX**	**AY**	**BX**	**BY**
**Safe**	**AX**								
	**AY**	.32*							
	**BX**	.09	-.11						
	**BY**	.24	.25*	.29*					
**Threat**	**AX**	.77**	.15	.18	.27*				
	**AY**	.41**	.73**	-.07	.29*	.25			
	**BX**	.30*	.02	.69**	.48**	.38**	.14		
	**BY**	.07	.21	.39**	.61**	.09	.27*	.47**	

**p* < .05, ***p* < .01, ****p* < .001

Table 8. Error rate correlations across conditions in the Stroop.

		Safe		Threat	
		**Congruent**	**Incongruent**	**Congruent**	**Incongruent**
**Safe**	**Congruent**				
	**Incongruent**	.28*			
**Threat**	**Congruent**	.72**	.23		
	**Incongruent**	.16	.78**	.07	

**p* < .05, ***p* < .01, ****p* < .001
